# Investigating the impact of stressful life events on neuroanatomy across adolescence

**DOI:** 10.64898/2026.05.06.723376

**Published:** 2026-05-08

**Authors:** Lisa Wiersch, Katharina Brosch, Erynn Christensen, Elvisha Dhamala

**Affiliations:** 1 Northwell, New Hyde Park, NY, USA; 2 Institute of Behavioral Science, Feinstein Institutes for Medical Research, Manhasset, USA; 3 Department of Psychiatry, Beth Israel Deaconess Medical Center, Harvard Medical School, MA, USA; 4 Department for Psychiatry, Donald and Barbara Zucker School of Medicine at Hofstra/Northwell, Uniondale, USA

## Abstract

Early-life stress elevates the risk of developing neuropsychiatric disorders. However, the mechanism underlying this vulnerability, and how they contribute to sex differences in these disorders, remain to be understood.

Here, we use multivariate brain-based predictive models to examine how the number, positive or negative appraisal, and impact of adolescent stressful life events reported either by the youth or their caregivers are reflected in neuroanatomy (cortical thickness, surface area, cortical and subcortical gray matter volume, and T1 intensity measures). We used data from the Adolescent Brain Cognitive Development (ABCD) study at 2-year (*N* = 6,301, age 11–12), 4-year (*N* = 5,000, age 13–14) and 6-year (*N* = 3,226, age 15–16) follow-up time points to examine the sex-independent and sex-specific neural correlates of stressful life events.

Our analyses showed mostly non-significant associations between stressful life events and neuroanatomy. However, we did find that the number of positively appraised stressful life events reported by the caregivers at the 4-year follow-up was significantly associated with cortical thickness, independent of sex, and with surface area in females only. Across three developmental timepoints, seven neuroanatomical measures, two reporting perspectives, and both sex-independent and sex-specific analyses, we show that the number, appraisal, and impact of stressful life events are largely not reflected in adolescent neuroanatomy.

## Introduction

Exposure to stressful life events during adolescence can increase vulnerability to neuropsychiatric disorders. Prior research predominantly focused on stress-processing regions rather than examining distributed effects across the whole brain, thereby limiting our understanding of how stress affects the developing brain as a whole. Furthermore, how subjective appraisal of stress and its impact can shape its effects, and whether these effects vary by sex or developmental stage is largely unknown. The present study addresses these gaps by employing a whole-brain multivariate analysis including multiple neuroanatomical measures to characterize how stressful life events are represented in the brain. We consider three timepoints, the subjective appraisal of stress and its impact, and sex-specific effects.

Stressful life events during adolescence, a time period characterized by high neural plasticity (Bethlehem et al., 2022; Gabard-Durnam & McLaughlin, 2020; Lockhart et al., 2018) and therefore a critical window of vulnerability, can disrupt neural maturation and the risk of developing a neuropsychiatric disorder (Lockhart et al., 2018; Sisk & Gee, 2022). So far, the majority of studies investigating the influence of stress on the brain have focused on the hypothalamic-pituitary-adrenal (HPA) axis and related brain regions, which are central to the brain’s stress response (Dedovic et al., 2009; McEwen et al., 2015; Pruessner et al., 2010). In response to stress, the pituitary gland triggers the adrenal glands to release cortisol, the stress hormone (Frodl & O’Keane, 2013). Modulating this core system are regions like the amygdala, which activates the HPA-axis (Barry et al., 2017; Herman et al., 2005; Pruessner et al., 2010), and the hippocampus, which provides inhibitory feedback (Frodl & O’Keane, 2013). Stress can alter neurochemistry, synaptic plasticity, neural activity, cytoarchitecture, and neurogenesis in these regions (Kim et al., 2015). Similarly, the prefrontal cortex, which typically inhibits the amygdala, is vulnerable to stress, which can result in structural remodeling of synaptic connections, impaired cognitive flexibility, and reduced functional connectivity (McEwen et al., 2016). Given the focus of previous studies on the HPA axis and its modulatory brain regions, it remains unclear whether stress affects brain regions beyond these regions. Univariate approaches focusing on single brain regions may miss complex, distributed responses across multiple brain regions. Therefore, whole-brain multivariate analyses are needed to characterize the distributed effects of stress. Machine learning techniques offer a powerful, data-driven framework to identify neural signatures distributed across multiple brain regions (Huberty & Morris, 1989).

Understanding whole-brain neural signatures of stress also requires an examination of whether subjective appraisal (i.e., whether it is experienced as a positive or negative event) of stress and its impact shapes its neural signatures. The same life event (e.g., relocation or parental divorce) may be objectively similar, but appraised differently across youth, potentially altering their neuroanatomical correlates. Several studies have linked stress to neuroanatomy using the Perceived Stress Scale (PSS, (Cohen et al., 1983)), which measures the degree to which situations are appraised as stressful (H. Li et al., 2014; Zimmerman et al., 2016). However, this scale does not capture the subjective appraisal of stress as positive or negative. Beyond the subjective appraisal, the subjective impact, (i.e., how much it affected a person’s life, regardless of valence) of a stressful life event may critically determine its neural signature. Ringwald et al. (2021) demonstrated that the total number of stressful life events, alongside with their cumulative impact was significantly associated with left medial orbitofrontal cortex volumes in adults, driven by both positive and negative event appraisals. The assessment of stressful life events, their appraisal, and impact in the developmental contexts may be complicated by the reporter perspective: youth and caregivers may differ in their reporting of which events occurred and in appraising their impact. Caregivers may also identify events unrecognized by youth (e.g. negative change in the parent’s financial situation) or perceive the appraisal and impact of an event differently than youth (e.g., considering a relocation to have had a limited, but positive impact on the youth, while the youth report it to have had a large, negative impact). It is therefore crucial to consider how the effects of stress on neuroanatomy may vary based on the reporter.

A critical gap in research is how stress and the appraisal of stress manifests not only in different regions of the brain, but also whether the neural signatures vary depending on the neuroanatomical measure. Previous studies investigating stress have predominantly focused on a single measure, but comprehensively uncovering the multivariate effects of stress necessitates investigating multiple measures, such as cortical thickness, surface area, gray matter volume, and T1-intensity. Cortical thickness is the distance between the gray-white matter junction and the pial surface (Fehlbaum et al., 2022). It rapidly increases during prenatal and neonatal development, peaks in early childhood, and subsequently declines throughout adolescence and adulthood (Bethlehem et al., 2022). Surface area is the area of the cortical outer layer (Fehlbaum et al., 2022). It continuously increases throughout childhood, reaching its peak in adolescence, and then gradually declines (Bethlehem et al., 2022). Grey matter volume is the total amount of neuronal tissue. Cortical grey matter volumes are estimated as the product of cortical thickness and surface area (Fehlbaum et al., 2022; Vijayakumar et al., 2016), whereas subcortical grey matter volumes refer to grey matter nuclei in the subcortex. Cortical volume increases throughout early childhood and peaks between early and late childhood, followed by a slow and steady decrease until late adulthood. In contrast, subcortical volumes show a slower, more prolonged increase, a later peak in adolescence and a slower decline (Bethlehem et al., 2022). T1-weighted intensity reflects the differential T1 relaxation times of brain tissue protons, creating inherent contrasts between grey and white matter tissues and facilitating the quantification of distinct anatomical structures, such as cortical and subcortical grey matter and white matter volumes (Serai, 2022). White matter consists of myelinated axons, essential for signal transmission throughout the brain. It matures throughout childhood and adolescence and peaks in young adulthood (Bethlehem et al., 2022; Lebel & Deoni, 2018). Considering the inherent differences and distinct developmental trajectories (Bethlehem et al., 2022) of the individual measures, it is crucial to analyze the neural signatures of stress in multiple neuroanatomical measures across time.

The neural signatures of stress are determined by multiple factors, with timing and sex being two particularly important determinants. The effects of stress on the brain can differ depending on when exposure occurs during sensitive developmental periods (Eiland & Romeo, 2013; Gee & Casey, 2015). Beyond timing, sex can influence stress processing and neurodevelopmental trajectories. Sex differences exist in the neuroendocrine response to stress in terms of the HPA-axis, genetics, epigenetics, and the valence of stress (Bangasser & Valentino, 2014; Bangasser & Wicks, 2017; Hodes et al., 2023). Moreover, males and females follow distinct neural developmental trajectories. Females reach peak gray matter volumes 1–2 years earlier than males and demonstrate higher subcortical volumes for e.g. the hippocampus after puberty. In contrast, males show steeper growth rates for white matter volumes and larger cortical surface area until the age of 15 (Gur & Gur, 2016; Koolschijn & Crone, 2013; Lenroot et al., 2007). Taken together, the development of the brain creates a time window of heightened vulnerability to stress dysregulation, with sex-specific trajectories emerging from different gonadal hormone influences (Bale & Epperson, 2015). Thus, given the complex influences of sex and timing on neurobiology and stress, it is highly important to consider both factors when characterizing the neural signatures of stress.

In the present study, we investigate the associations between stressful life events and brain structure across three timepoints during adolescence using brain-based predictive models leveraging data from the Adolescent Brain and Cognitive Development (ABCD) dataset. Specifically, we examine how the number, appraisal, and impact of stressful life events relate to different neuroanatomical measures (cortical thickness, surface area, cortical and subcortical grey matter volumes and T1-intensity measures) at the 2-year (ages 11–12), 4-year (ages 13–14) and 6-year (ages 15–16) follow-up timepoints. To capture potential sex-specific effects, we examine these relationships separately for females and males. Furthermore, we computed separate models for youth- and caregiver-reported stressful life events to account for potential discrepancies between self-report and external observations.

## Methods

### Dataset

The ABCD study is a prospective longitudinal study assessing children starting at ages 9–10 and following participants for 10 years (Casey et al., 2018). The study recruited participants from 21 research sites across the United States comprising a cohort of nearly 12,000 participants. Participants complete a comprehensive battery of neuroimaging, behavioral, developmental, and psychiatric assessments. Here, we used the neuroimaging data and assessments of stressful life events based on the Adverse Life Events Scale of the PhenX Toolkit from the 2-year (ages 11–12), 4-year (ages 13–14), 6-year (ages 15–16) follow-up time points from the ABCD 6.0 release. We included subjects with complete and quality-controlled neuroimaging data, as well as complete demographic and life events data relevant for our analyses (see Figure S1-S3 for details of the inclusion pipelines). We also randomly excluded members of the same family to ensure no siblings were included in the sample. Overall, we included 6,301 subjects at 2-year follow-up (2,913 females), 5,000 subjects at 4-year follow-up (2,327 females), 3,226 subjects at 6-year follow-up (1,519 females). Demographic information for each timepoint is reported in Table S1.

### Neuroimaging data

Structural T1-weighted neuroimaging data were collected using 3T MRI scanners. The neuroimaging protocol and parameters for the acquisition of T1-weighted images have been detailed in prior work (Casey et al., 2018; Hagler et al., 2019). We extracted total surface area, total grey matter volume, mean cortical thickness, and mean T1-intensity in grey and white matter from 74 bilateral cortical regions (148 total) defined by the Destrieux Atlas (Destrieux et al., 2010). We also included subcortical grey matter volumes and T1-intensity measures covering 19 regions (bilaterally: accumbens, amygdala, cerebellar cortex, caudate, hippocampus, pallidum, putamen, thalamus, ventral diencephalon, unilaterally: brainstem). To ensure generalizability, we repeated our cortical analyses using the Desikan-Killiany Atlas (Desikan et al., 2006), which includes 34 bilateral cortical regions (68 total). All neuroanatomical measures were provided by the ABCD study’s standard FreeSurfer preprocessing pipeline.

### Stressful life events questionnaire

The questionnaire assessing stressful life events in the ABCD study is based on the Adverse Life Events Scale of the PhenX Toolkit (PhenX Toolkit, *Life Events - Child*. https://www.phenxtoolkit.org/protocols/view/211501). The scale consists of 25 items administered directly to the child (youth-report) or a proxy (caregiver-report) covering various stressful events (e.g., injuries, death, crime, illness, relocations, new siblings) experienced during the previous year. The respondent reviews the list of items and indicates which events have occurred. If endorsed, follow-up questions are asked about timing, appraisal, and impact (“Did this happen in the last year?”; “Was this a good or a bad experience?”/”Was this a good or a bad experience for your child?”; “How much did the event affect you?”/”How much did the event affect your child?”). After the initial administration based on the 25 items, some items were added at later time points and are thus unavailable at earlier waves (e.g., school shootings, homelessness, deportation, parental hospitalization, foster care, and witnessing violence; detailed description of items added at later timepoints: https://docs.abcdstudy.org/latest/documentation/non_imaging/mh.html#mh_y_ple).

We focused on six summary variables: (1) the **number of total stressful life events** (count of endorsed events), (2) the **impact of total stressful life events** (sum of impact ratings for all endorsed events), (3) the **number of positively appraised events** (count of endorsed events rated as “good”), (4) the **impact of positively appraised events** (sum of impact ratings for endorsed events rated as “good”), (5) the **number of negatively appraised events** (count of endorsed events rated as “bad”), (6) the **impact of negatively appraised events** (sum of impact rating for endorsed events rated as “bad”). Data for these six summary variables were collected from either the youth or a caregiver. We only included participants where data from both sources were available, resulting in each of the six summary variables being represented twice (once from the youth-report and once from the caregiver-report) for a total of 12 variables. Including data collected across three different timepoints, the stressful life events are assessed cumulatively, as earlier occurrences are reflected in subsequent assessments.

### Descriptive analyses

To assess potential differences in number or impact of differently appraised events, we ran t-tests contrasting positively and negatively appraised events. Similarly, we examined sex differences in the summary variables using t-tests, contrasting all 12 variables between males and females. To evaluate similarity between youth- and caregiver-report, we calculated correlation coefficients for the six summary variables.

### Brain-Based Predictive Modeling

**Figure 1.**
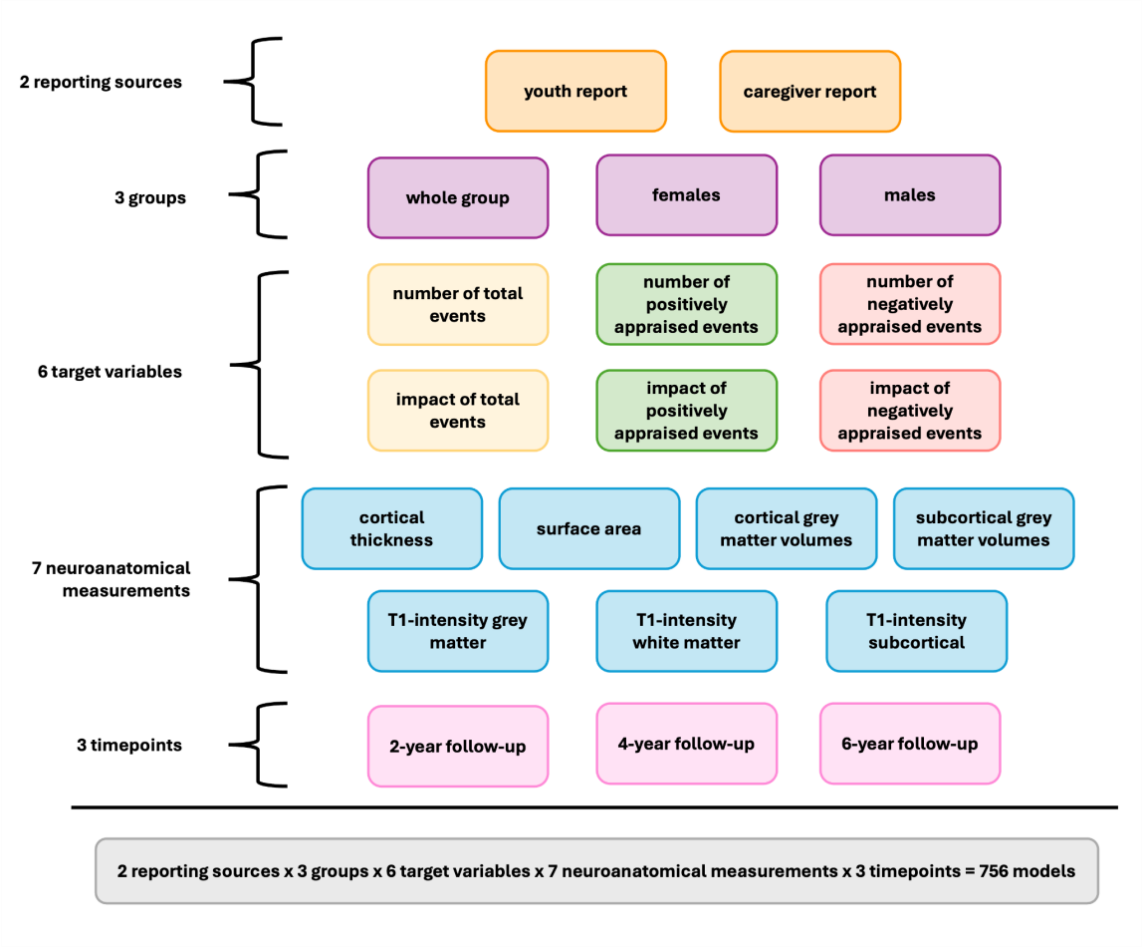
Analysis overview

We used a cross-validated brain-based predictive modeling framework to establish associations between neuroanatomical measures and stressful life events. This framework minimizes the risk of overfitting and prevents data leakage, while capturing robust and interpretable associations between neuroimaging-derived measures and phenotypic data (Dhamala, Yeo, et al., 2023). Here, we used linear ridge regression to predict the summary variables based on regional neuroanatomical measures. We trained separate models for each combination of neuroanatomical measure and stressful life event variable at each timepoint. Additionally, we developed sex-independent and sex-specific models to explore potential sex-specific associations between stressful life events and neuroanatomy. Overall, the combination of 7 neuroanatomical measures, 12 target variables (6 youth-report and 6 caregiver-report summary stress variables) and 3 groups (sex-independent group (whole group) and sex-specific groups (females only and males only)) and three time points led to 756 total models (Figure 1).

For each model, we performed 100 iterations of a random train-test (80%−20%) split. For each iteration, participants from the same site were not split across train/test sets to account for potential variability introduced by the imaging site. Within each train-test split, we optimized the regularization hyperparameter using 5-fold cross validation, ensuring that participants from a given site were not split across folds. After hyperparameter optimization in the training set, the model was fit to the training data using the optimizing hyperparameter and evaluated on the corresponding test set. Model performance was assessed using prediction accuracy, defined as the correlation between the predicted values and the observed values, consistent with prior work (Chen et al., 2022, 2023; Dhamala, Rong Ooi, et al., 2023; J. Li et al., 2019). The 100 iterations of the train-test split resulted in a distribution of prediction accuracy values for each set of models.

To evaluate the significance of each set of models, we compared the distribution of prediction accuracy values to corresponding distributions of null models. Null models were generated by randomly permuting the target variable (respective stressful life events summary variable) within each site and using the permuted data to train and test models using the optimized hyperparameters. This process was repeated for 1000 train-test splits, generating a null distribution of accuracy values. We calculated p-values for model performance as the proportion of null models with prediction accuracies greater than or equal to the mean of the original model’s accuracy.

We corrected p-values for multiple comparisons across the 12 target variables for the respective groups (whole group, males only, females only) individually using the Benjamini-Hochberg False Discovery Rate procedure (Benjamini & Hochberg, 1995). Results were considered statistically significant if p_FDR_ ≤ 0.050.

### Feature weights

To increase interpretability and reliability, we transformed the feature weights obtained from the models using the Haufe transformation (Haufe et al., 2014). We then calculated mean feature importance for each set of models. We assessed similarity between feature weights of models using Pearson correlation.

All analyses were conducted in Python (3.12.4) using Jupyter Notebook and visualizations were generated using the seaborn package.

## Results

### Descriptive analyses

#### 2-year follow-up

At the 2-year follow-up, for all three groups (whole group, males only, and females only) the number of negatively appraised events was greater than the number of positively appraised events, and the impact of negatively appraised events was greater than the impact of positively appraised events for the youth and the caregiver report (see Table S2, and S3 for statistics).

**Figure 2.**
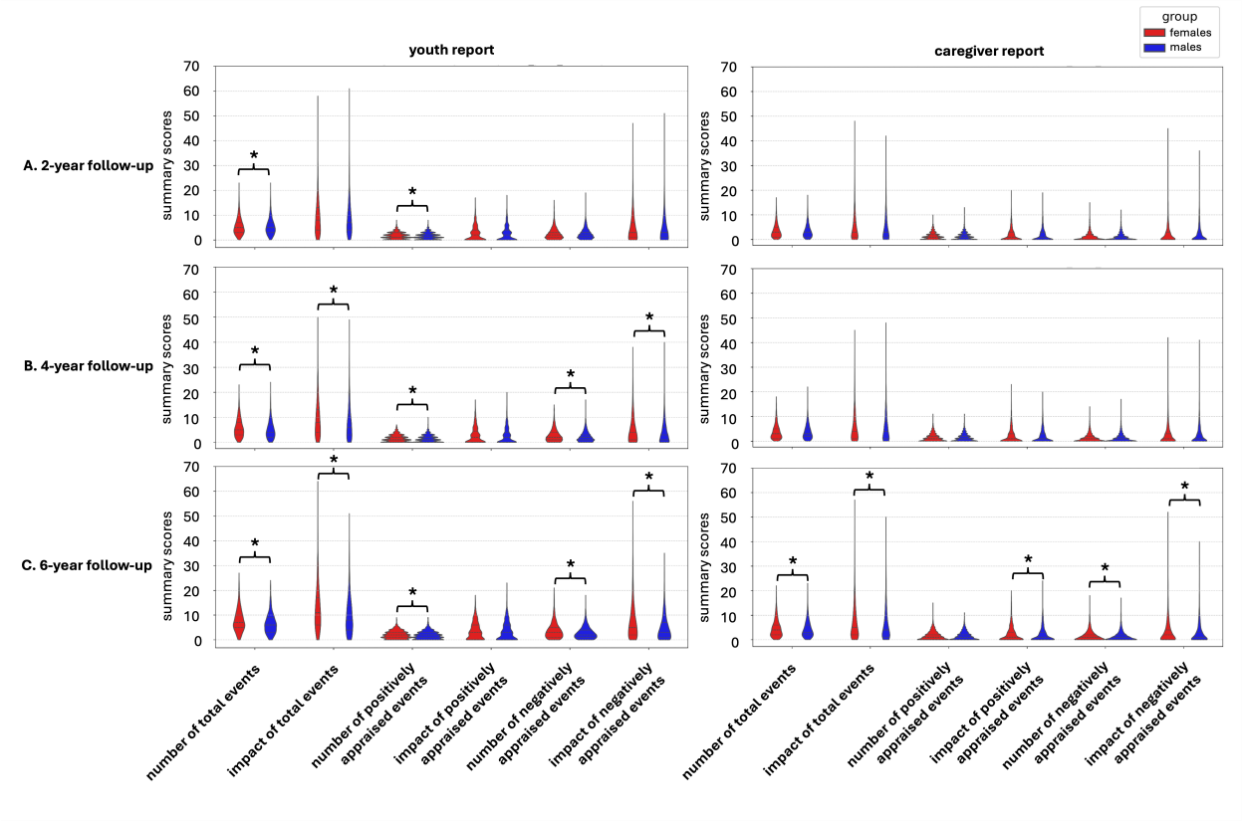
Sex differences in the distribution of summary variables. Descriptive statistics and t-tests contrasts of sex-specific groups for stressful life events variables at the (A) 2-year follow-up, (B) 4-year follow-up, (B) 6-year follow-up timepoint for the youth report and caregiver report. Asterisks indicate a significant difference.

Comparing across the sexes, for the youth report, females reported more positively appraised events than males. All other sex comparisons were not significant (Figure 2A).

Correlating the summary scores between the youth and the caregiver report revealed weak to moderate, but significant, correlation for all scores (Table S4).

#### 4-year follow-up

At the 4-year follow-up, for all three groups, the number of negatively appraised events was greater than the number of positively appraised events, and the impact of negatively appraised events was greater than the impact of positively appraised events for the youth and the caregiver report (Table S2 and S3).

Comparing across the sexes, for the youth report, females reported a significantly higher number of total, positively, and negatively appraised events, and impact of total and negatively appraised events. Comparisons for the caregiver report did not reveal significant sex differences (Figure 2B).

Correlating the summary scores between the youth and the caregiver report revealed weak to moderate, but significant correlations for all scores (Table S4).

#### 6-year follow-up

At the 6-year follow-up, for all three groups, the number of negatively appraised events was greater than the number of positively appraised events, and the impact of negatively appraised events was greater than the impact of positively appraised events for the youth report. For the caregiver report, males reported a significantly higher number of positively appraised events compared to negatively appraised events, but this was not seen in females or when examining the whole group together. The impact of negatively appraised events was significantly greater than the impact of positively appraised events in all three groups for the caregiver report (Table S2 and S3).

Comparing across the sexes, for the youth report, females reported significantly higher number of total, positively, and negatively appraised events, and impact of total and negatively appraised events. For the caregiver report, females reported a higher number and impact of negatively appraised events, and the impact of total events (Figure 2C).

Correlating the summary scores between the youth and the caregiver report revealed weak to moderate, but significant correlations for all scores (Table S4).

### Brain-Based Predictive Models

We used brain-based predictive models to capture associations between neuroanatomy and the number, appraisal and impact of stressful life events at three different timepoints. We trained separate models based on cortical thickness, surface area, cortical or subcortical grey matter volumes, and T1-intensity measures.

#### 2-year follow-up

**Figure 3.**
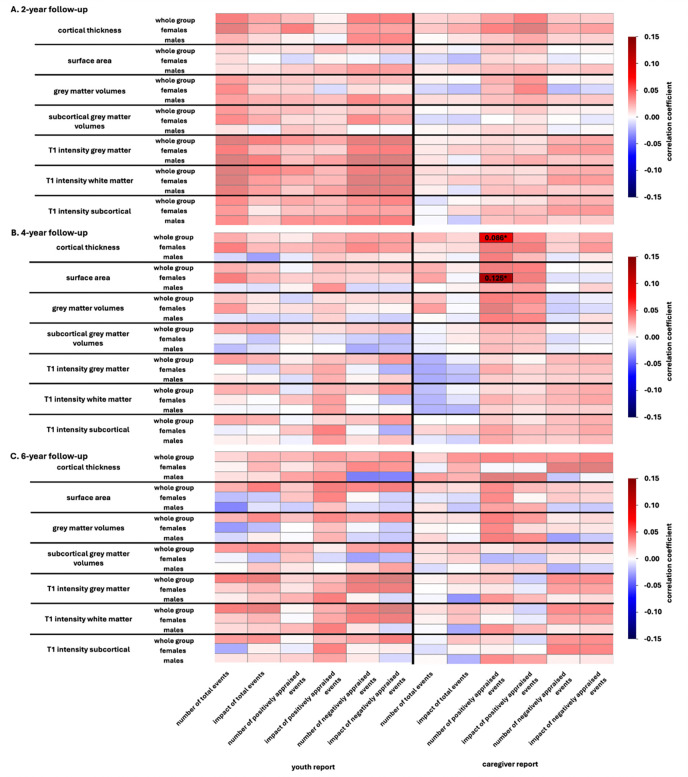
Predictive Performance of neuroanatomical measures for stressful life event outcomes at 2-year, 4-year, and 6-year follow-up. This heatmap illustrates the mean prediction accuracy for models trained to predict the number, appraisal, or impact of stressful life events based on different neuroanatomical measures (cortical thickness, surface area, cortical and subcortical grey matter volumes and T1-intensity measures). The colors of the heatmap indicate the strength of the association. Negative associations are depicted in blue, positive associations are depicted in red. Cortical measures of cortical thickness, surface area, grey matter volumes and T1-intensity are based on the Destrieux parcellation. Non-significant associations are depicted with reduced opacity. Significant predictions are labeled with their prediction accuracy.

At the 2-year follow-up, our brain-based predictive models did not accurately predict any of the summary stressful life events variables from the youth or caregiver report data. The consistent non-significant results for the sex-independent and sex-specific models, despite the high sample sizes, suggest that stressful life events are not necessarily reflected in the neuroanatomy of 11–12 year-olds. Results for all groups are in Figure 3A and accuracy values are in Figure S4.

#### 4-year follow-up

At the 4-year follow-up, our brain-based predictive models yielded two significant predictions (Figure 3B, Figure S5).

**Figure 4.**
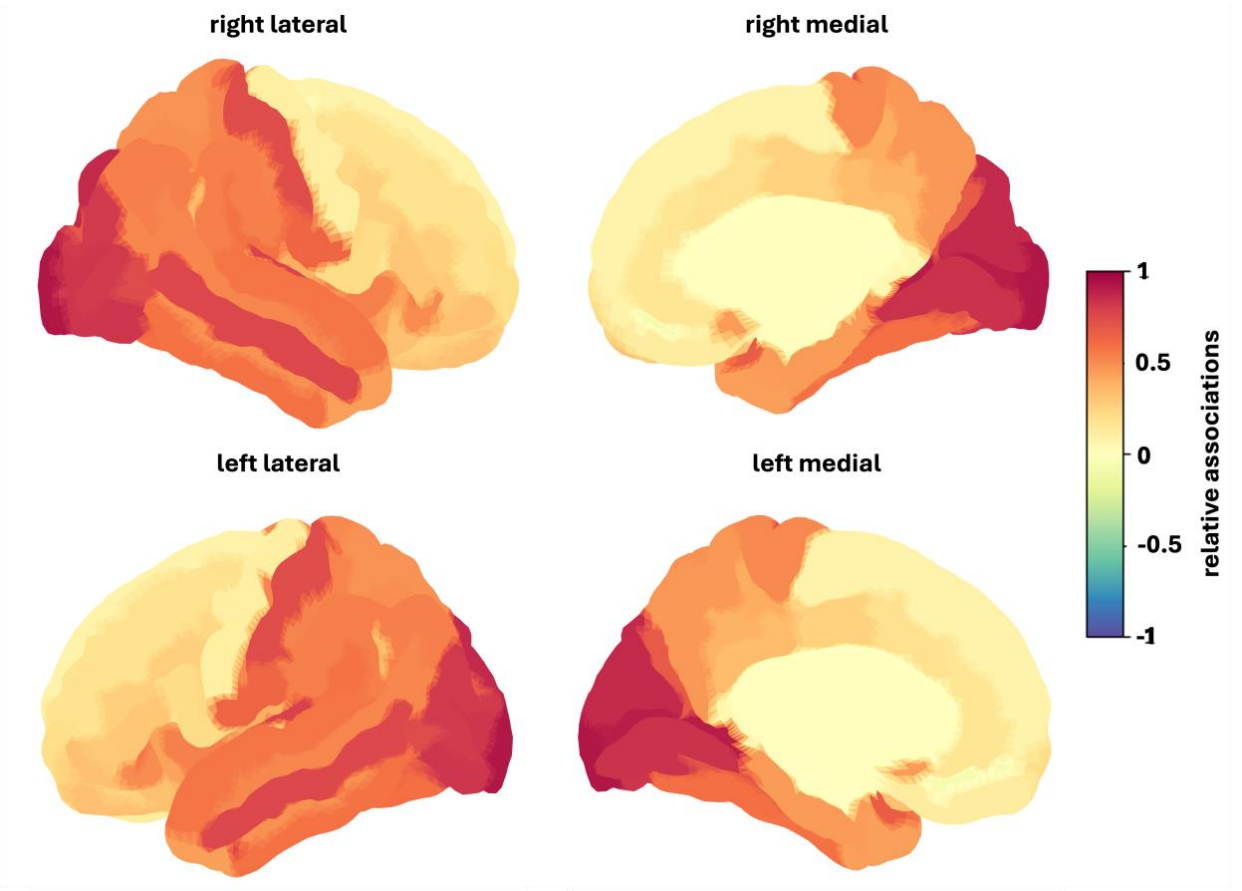
Relative associations between cortical thickness and number of positively appraised stressful life events for the whole group. Red colors indicate stronger positive associations, blue colors indicate stronger negative associations. To facilitate visualization, association values were divided by the maximum value of this individual prediction.

The sex-independent analyses revealed that cortical thickness significantly predicted the number of positively appraised events based on the caregiver report data (r = 0.086, p_FDR_ = 0.012) for the whole group. These associations were predominantly positive, with the strongest positive associations in the right occipital pole, inferior and superior occipital gyrus and calcarine sulcus (Figure 4).

**Figure 5.**
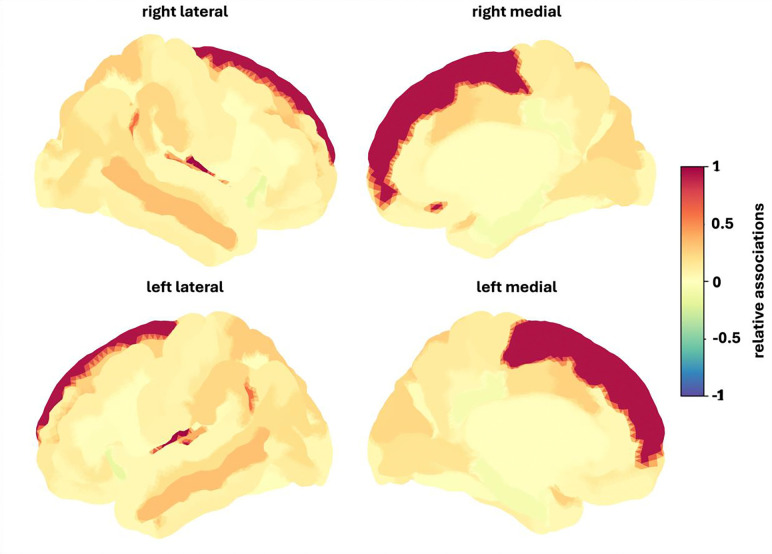
Relative associations between surface area and number of positively appraised stressful life events for females. Red colors indicate stronger positive associations, blue colors indicate stronger negative associations. To facilitate visualization, association values were divided by the maximum value of this individual prediction

The sex-specific analyses revealed that surface area significantly predicted the number of positively appraised events based on the caregiver report data (r = 0.125, p_FDR_ = 0.024) in females. These associations were also generally positive, with the strongest positive associations in the superior frontal gyrus, the left middle frontal gyrus and left superior temporal sulcus (Figure 5).

Feature weights from these two models were not correlated (r = −0.147, p = 0.072), indicating they map onto unique neuroanatomical regions.

Results for all groups are in Figure 2B and accuracy values are in Figure S5.

#### 6-year follow-up

At the 6-year follow-up, the brain-based predictive models did not predict any of the summary variables for youth or caregiver report based on neuroanatomical measures. Results for all groups are in Figure 3C and accuracy values are in Figure S6.

The consistent lack of significant predictions by the brain-based predictive models at the 6-year follow-up demonstrates that stress is not reflected in neuroanatomy in 15–16 years olds.

All analyses based on the Destrieux parcellation were repeated using the Desikan-Killiany parcellation. Across all timepoints, none of the predictive models yielded significant results after correction for multiple comparisons (Figure S7, Figure S8, and Figure S9, respectively).

## Discussion

Here, we used brain-based predictive models to investigate the associations between stressful life events and neuroanatomy. In a comprehensive analysis, we assessed 6 target variables capturing number, appraisal, and impact of stressful life events based on 2 reporting sources in 7 neuroanatomical measures in sex-independent and sex-specific groups across three timepoints. These combinations led to 756 analyses in total, with 754 non-significant associations and only two significant associations after correcting for multiple comparisons. Overall, our findings suggest that the cross-sectional influences of stressful life events on neuroanatomy may not be easily predictable.

The fact that the majority of our models did not yield significant results contrasts with extant literature investigating the impact of stress on the brain, often reporting effects in subcortical structures such as the hippocampus, amygdala, and prefrontal cortex (Kim et al., 2015; McEwen et al., 2016; Zhang et al., 2018). This discrepancy may stem from different operationalizations of stress. Studies have often operationalized stress based on physiological markers, such as basal glucocorticoid release or hyperactivation of the HPA axis, leading to elevated glucocorticoid levels (McEwen et al., 2016; Zhang et al., 2018). Here, we examined the frequency, appraisal, and impact of stressful events as reported by the youth or their caregivers, which reflects naturalistic stress experiences in youth’s daily lives. Another explanation for the discrepancy between our results and previous literature are the differences in analytical approaches. While previous studies predominantly used univariate approaches to examine individual brain regions, we used a multivariate approach focusing on the whole brain. A univariate effect of stress on the brain can be found by contrasting two groups. In the case of the present study, multivariate analyses test how accurately a certain number operationalized as the number, appraisal, or impact of stressful life events can be predicted on the individual level. A significant finding by one approach and not the other is not mutually exclusive. While localized associations between specific events and brain regions may exist, our whole-brain multivariate approach - which examined multiple neuroanatomical measures and target variables - did not yield significant predictive associations across most of our analyses after correcting for multiple comparisons

Out of the 756 models, two yielded significant associations at the 4-year follow-up based on the caregiver report data. The number of positively appraised events was positively linked to cortical thickness across the whole group, and to surface area in females, suggesting a higher number of events is associated with greater thickness and area, respectively. However, given that both significant findings were exclusively linked with positively appraised stressful life events, it could be argued that these results do not represent stressful events in the traditional sense. Moreover, the two significant predictions were exclusively based on the caregiver-reported data, meaning that the significant associations do not directly represent the lived experience of the child. Although the correlation between youth and caregiver report for the number of positively appraised events at the 4-year follow-up was significant, the correlation coefficient itself was rather weak (Table S4). While this does not necessarily validate caregiver appraisal as more accurate representations of youth’s stressful life events, it could be speculated that the significant associations do not reflect the lived experience of a child. Instead, they potentially reflect the environment shaped by events that caregivers appraise as positive for the youth, which in turn may be reflected in neuroanatomy.

For cortical thickness, the right occipital pole, inferior and superior occipital gyrus and the calcarine sulcus exhibited the strongest relationships. Studies investigating the association between these brain areas and stress are relatively limited and predominantly focus on Post-Traumatic Stress Disorders (PTSD), consistently reporting reductions in cortical volume in individuals with PTSD compared to healthy controls in occipital areas (Chao et al., 2012; Cwik et al., 2020; Tavanti et al., 2012). The findings of our study show a complementary effect in terms of positively appraised events being associated with higher cortical thickness. This aligns with Kahl et al. (2020), who found a positive association between trait and resilience, measured via self-report, and cortical thickness and occipital and temporal regions, suggesting that positive psychological orientations toward stress more broadly may be reflected in occipital neuroanatomy.

For surface area, the bilateral superior frontal gyrus, left middle frontal gyrus and left superior temporal sulcus exhibited the strongest relationships. The structural volumes of the superior frontal gyrus are associated with reappraisal ability (i.e., reducing the personal relevance or emotional impact of negative events, (Falquez et al., 2014)). Similarly, higher levels of stress are associated with greater neural activity in the left superior frontal gyrus, a core region for cognitive control and emotion-regulation processes (Wang et al., 2019). The middle frontal gyrus also plays a role in appraised stress, as Michalski et al. (2017) reported a positive association of regional cortical thickness with appraised stress and sadness and a negative association with positive effect. Finally, limited studies have examined the role of superior temporal sulcus in stress, but the region is a hub for social processing, perception, cognition, involved in understanding the actions, mental states and language of others (Deen et al., 2015; Redcay, 2008). Altogether, the regions that exhibited the strongest relationships with prediction of positively appraised events are involved in broader perceptual processes, including social perception, stress perception, or stress appraisal. This suggests that these regions serve as a common neural substrate for the evaluation and interpretation of diverse stimuli in the appraisal process. Notably, associations were only observed in females, even though no significant sex difference for the number of positively appraised events was reported (Figure 2B). Overall, our results demonstrate that the influences of stressful life events on neuroanatomy are generally subtle and not easily predictable yet may still possess sex-specific characteristics.

Importantly, while most of our results yielded non-significant associations between stressful life events and neuroanatomy using a multivariate approach, these findings do not negate prior work showing significant effects of stress on the brain. Instead, the associations of frequency, appraisal, and impact of stressful events might be masked by ongoing developmental changes during adolescence or the neural characterization manifested later in life until adulthood, which is outside of the time window captured in the present analyses. Another consideration is that the neural characterizations might manifest in a different brain modality: While the present study extensively analyzes neuroanatomical measures, prior research utilizing ABCD data, has demonstrated that adverse life events are associated with decreased functional connectivity between cortical and subcortical regions (Elton et al., 2025). Additionally, our operationalization of stress may not adequately capture the heterogeneous nature of environmental stressors youth are exposed to. Other environmental adversity-related factors, including neighborhood threat, scarcity, were demonstrated to be reflected in cortical thickness (Brosch et al., 2025). Importantly, the timing of adversity exposure during development critically determines its neural targets, with early-life adversity predominantly affecting sensorimotor networks and later-life adversity predominantly affecting association networks (Michael et al., 2025). Furthermore, adversity can dynamically influence neuroplasticity, by accelerating or delaying developmental trajectories, and by amplifying or dampening their magnitude, depending on the timing. Overall, the interactions between adverse experiences, neuroplasticity, and developmental timing can shape the neural responses to adversity events, underscoring the need for a nuanced investigative framework.

It is important that our findings are interpreted in the context of some key limitations. First, our operationalization of stress was limited to specific events captured by the Adverse Life Events scale, which does not fully capture all stress-related factors. Furthermore, demographic variables such as socioeconomic status can introduce a challenge for isolating the neuroanatomical correlates of stress, given its intricate relationship with stressful life events (e.g. homelessness, eviction). Second, while our analyses examined multiple neuroanatomical measures and stress summary scores, we exclusively used linear ridge regression and we did not test other algorithms, which potentially yield different results. Third, while the ABCD study is a large-scale and heterogeneous dataset collected at multiple sites across the U.S., its exclusive focus on the U.S. may limit the generalizability of our results across international populations.

Overall, we assessed the neuroanatomical basis of the frequency, appraisal, and impact of stressful life events using a multivariate brain-based predictive modeling approach covering multiple timepoints during development. Our results yielded predominantly non-significant associations indicating that stressful life events are largely not reflected in neuroanatomy. This may reflect the complexity of adversity exposure as diverse stressors likely exert differentiated effects on brain structure. Therefore, multi-dimensional assessments that integrate multiple stressor types and environmental contexts are necessary to accurately characterize the neuroanatomical impact of adversity.

## Supplementary Material

Supplement 1

## Data Availability

Data used in the preparation of this article were obtained from the Adolescent Brain Cognitive Development™ (ABCD) Study, held in the NIH Brain Development Cohorts Data Sharing Platform. This is a multisite, longitudinal study designed to recruit more than 10,000 children aged 9–10 and follow them over 10 years into early adulthood. The ABCD Study^®^ is supported by the **National Institutes of Health** and additional federal partners under award numbers: U01DA041048, U01DA050989, U01DA051016, U01DA041022, U01DA051018, U01DA051037, U01DA050987, U01DA041174, U01DA041106, U01DA041117, U01DA041028, U01DA041134, U01DA050988, U01DA051039, U01DA041156, U01DA041025, U01DA041120, U01DA051038, U01DA041148, U01DA041093, U01DA041089, U24DA041123, U24DA041147. A full list of supporters is available at Federal Partners - ABCD Study. ABCD Consortium investigators designed and implemented the study and/or provided data but did not necessarily participate in the analysis or writing of this report. This manuscript reflects the views of the authors and may not reflect the opinions or views of the NIH or ABCD Consortium investigators. The ABCD data repository grows and changes over time. The ABCD data used in this report came from the ABCD 6.0 release.

## Code availability

The code for data preparation, model training and computation of further analyses if available on Gitlab: https://gitlab.com/lwiersch/investigating-the-impact-of-stressful-life-events-on-neuroanatomy-across-adolescence
