# Supplementary material for: Investigating the impact of stressful life events on neuroanatomy across adolescence
Source: bioRxiv. 2026 May 8:2026.05.06.723376. Preprint. [Version 1] doi: 10.64898/2026.05.06.723376 (PMC13174451; doi:10.64898/2026.05.06.723376)
Supplement: Supplement 1 [file media-1.docx]

**SUPPLEMENTARY INFORMATION**

**Investigating the impact of stressful life events on neuroanatomy across adolescence**

Wiersch et al.

**Supplementary Methods**

**
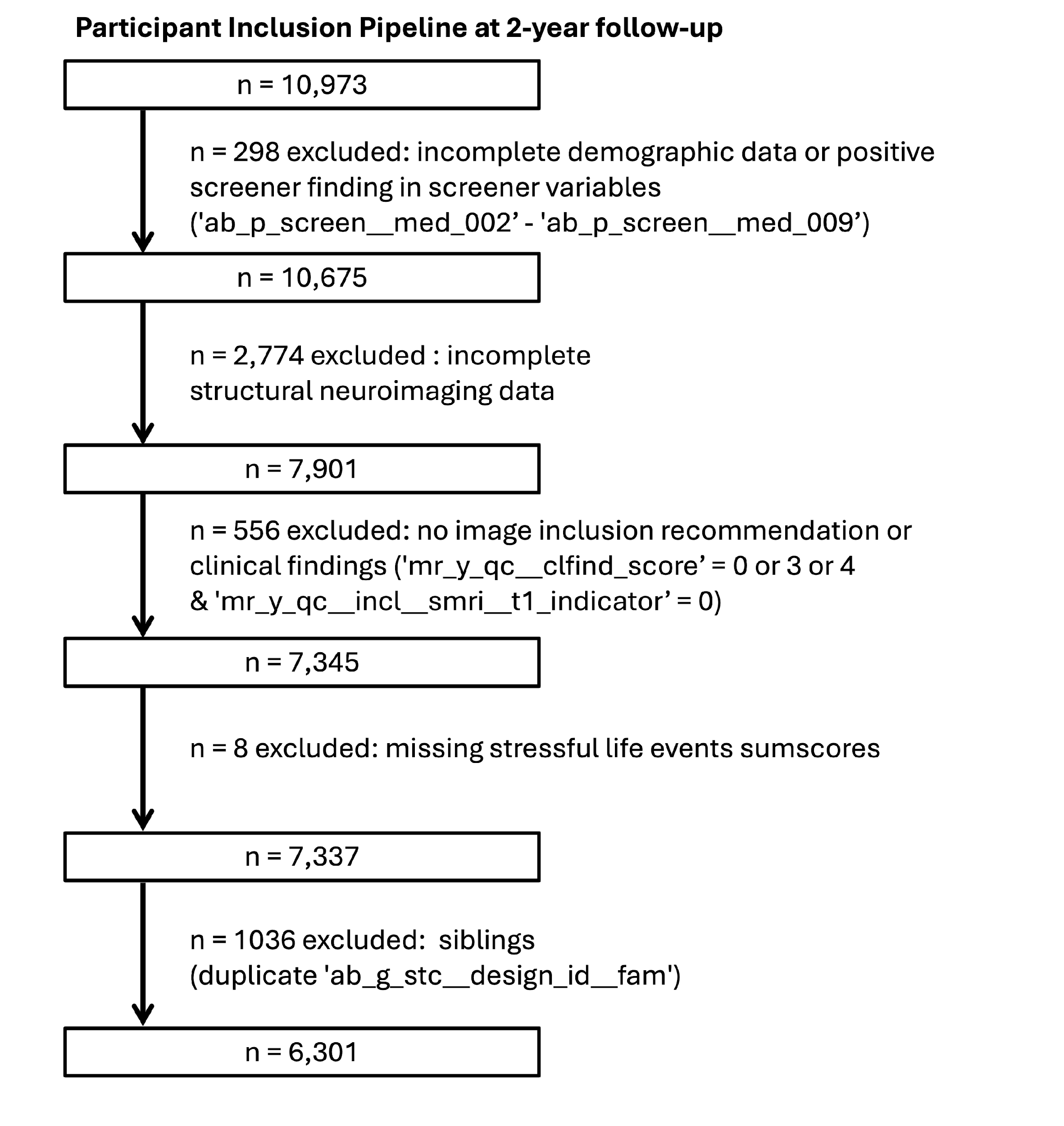
**

**Supplementary Figure 1. Participant Inclusion and exclusion workflow at 2-year follow-up.**

**
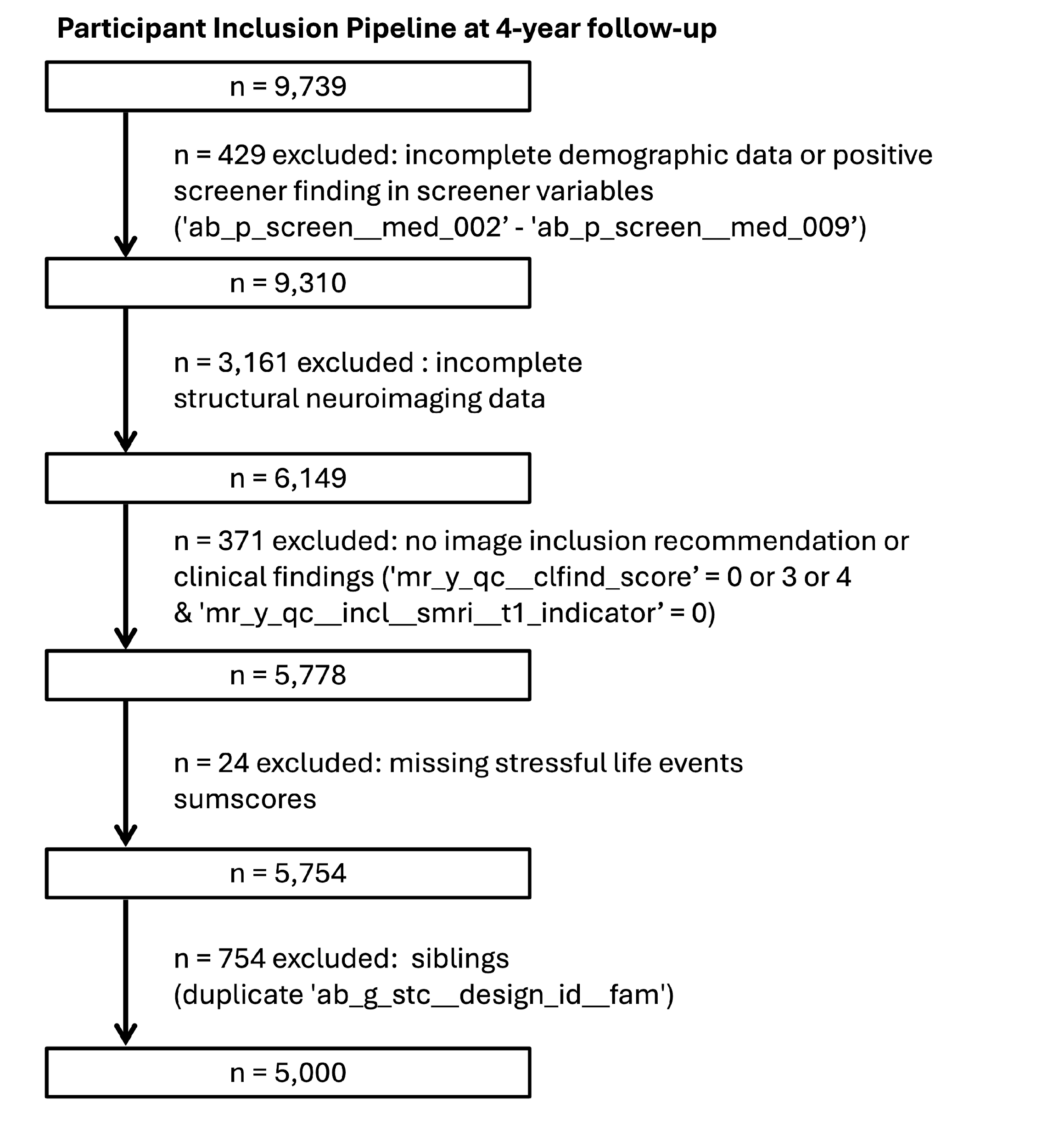
**

**Supplementary Figure 2. Participant Inclusion and exclusion workflow at 4-year follow-up.**

**
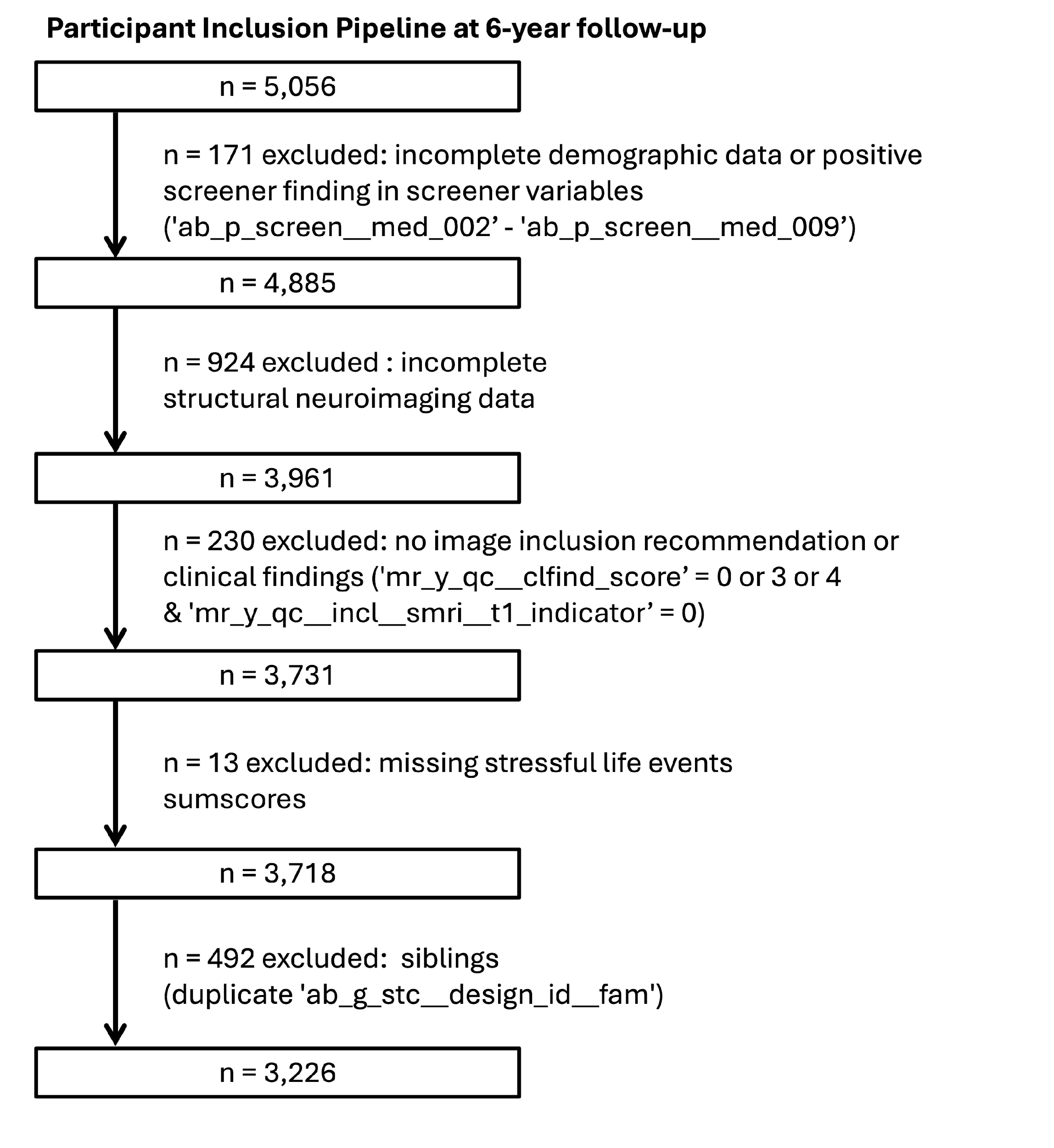
**

**Supplementary Figure 3. Participant Inclusion and exclusion workflow at 6-year follow-up.**

**Supplementary Table 1. Demographic information**Demographic information (age, ethnicity, and income) for all subjects included for the descriptive and predictive analyses. Demographic information is reported separately for males and females for the 2-year (A), 4-year (B), and 6-year follow-up (C). Reported proportions (%) may not sum to exactly 100% due to rounding.

| **A) 2-year follow-up (*N* = 6,301)** |  |  |
| --- | --- | --- |
|  | **males** | **females** |
| **Sample Size (%)** | 3388 (53.77%) | 2913 (46.23%) |
| **Age (mean, SD)** | 12.01 (0.65) | 11.96 (0.65) |
| **Ethnicity** |  |  |
| Asian | 60 (1.77%) | 51 (1.75%) |
| Black | 456 (13.46%) | 413 (14.18%) |
| Hispanic | 675 (19.92%) | 610 (20.94%) |
| White | 1883 (55.58%) | 1518 (52.11%) |
| Other | 314 (9.27%) | 321 (11.02%) |
| **Income Category (count, %)** |  |  |
| Less than $5,000 | 92 (2.72%) | 79 (2.71%) |
| $5,000 through $11,999 | 109 (3.22%) | 68 (2.33%) |
| $12,000 through $15,999 | 69 (2.04%) | 56 (1.92%) |
| $16,000 through $24,999 | 111 (3.28%) | 96 (3.30%) |
| $25,000 through $34,999 | 168 (4.96%) | 167 (5.73%) |
| $35,000 through $49,999 | 221 (6.52%) | 222 (7.62%) |
| $50,000 through $74,999 | 416 (12.28%) | 386 (13.25%) |
| $75,000 through $99,999 | 431 (12.72%) | 386 (13.25%) |
| $100,000 through $199,999 | 1077 (31.79%) | 881 (30.24%) |
| $200,000 and greater | 435 (12.84%) | 357 (12.26%) |
| Don't know | 128 (3.78%) | 126 (4.33%) |
| Decline to answer | 131 (3.87%) | 89 (3.06%) |

| **B) 4-year follow-up (*N* = 5000)** |  |  |
| --- | --- | --- |
|  | **males** | **females** |
| **Sample Size (%)** | 2673 (53.46%) | 2327 (46.54%) |
| **Age (mean, SD)** | 14.15 (0.70) | 14.15 (0.72) |
| **Ethnicity** |  |  |
| Asian | 46 (1.72%) | 51 (2.19%) |
| Black | 367 (13.73%) | 369 (15.86%) |
| Hispanic | 553 (20.69%) | 486 (20.89%) |
| White | 1444 (54.02%) | 1173 (50.41%) |
| Other | 263 (9.84%) | 248 (10.66%) |
| **Income Category (count, %)** |  |  |
| Less than $5,000 | 58 (2.17%) | 55 (2.36%) |
| $5,000 through $11,999 | 64 (2.39%) | 49 (2.11%) |
| $12,000 through $15,999 | 43 (1.61%) | 43 (1.85%) |
| $16,000 through $24,999 | 92 (3.44%) | 70 (3.01%) |
| $25,000 through $34,999 | 132 (4.94%) | 115 (4.94%) |
| $35,000 through $49,999 | 161 (6.02%) | 164 (7.05%) |
| $50,000 through $74,999 | 291 (10.89%) | 242 (10.40%) |
| $75,000 through $99,999 | 319 (11.93%) | 278 (11.95%) |
| $100,000 through $199,999 | 841 (31.46%) | 750 (32.23%) |
| $200,000 and greater | 446 (16.69%) | 370 (15.90%) |
| Don't know | 112 (4.19%) | 85 (3.65%) |
| Decline to answer | 114 (4.26%) | 106 (4.56%) |

| **C) 6-year follow-up (*N* = 3226)** |  |  |
| --- | --- | --- |
|  | **males** | **females** |
| **Sample Size (%)** | 1707 (52.91%) | 1519 (47.09%) |
| **Age (mean, SD)** | 16.08 (0.65) | 16.06 (0.64) |
| **Ethnicity** |  |  |
| Asian | 34 (1.99%) | 21 (1.38%) |
| Black | 182 (10.66%) | 171 (11.26%) |
| Hispanic | 328 (19.21%) | 310 (20.41%) |
| White | 1010 (59.17%) | 852 (56.09%) |
| Other | 153 (8.96%) | 165 (10.86%) |
| **Income Category (count, %)** |  |  |
| Less than $5,000 | 34 (1.99%) | 25 (1.65%) |
| $5,000 through $11,999 | 28 (1.64%) | 27 (1.78%) |
| $12,000 through $15,999 | 23 (1.35%) | 17 (1.12%) |
| $16,000 through $24,999 | 51 (2.99%) | 22 (1.45%) |
| $25,000 through $34,999 | 68 (3.98%) | 62 (4.08%) |
| $35,000 through $49,999 | 91 (5.33%) | 93 (6.12%) |
| $50,000 through $74,999 | 153 (8.96%) | 135 (8.89%) |
| $75,000 through $99,999 | 190 (11.13%) | 189 (12.44%) |
| $100,000 through $199,999 | 598 (35.03%) | 546 (35.94%) |
| $200,000 and greater | 355 (20.80%) | 312 (20.54%) |
| Don't know | 48 (2.81%) | 40 (2.63%) |
| Decline to answer | 68 (3.98%) | 51 (3.36%) |

**Supplementary Results**

**Table S2.** Descriptive statistics of the whole group for the 2-year follow-up, 4-year follow-up, and 6-year follow-up timepoint for the youth report and caregiver report.

|  |  | **youth report** | | | | | |
| --- | --- | --- | --- | --- | --- | --- | --- |
| **2-year follow-up** |  | **number of total events** | **impact of total events** | **number of positively appraised events** | **impact of positively appraised events** | **number of negatively appraised events** | **impact of negatively appraised events** |
|  | **mean** | 5.36 | 1.57 | 2.43 | 8.80 | 2.50 | 4.96 |
|  | **std** | 3.27 | 1.33 | 2.24 | 6.98 | 2.87 | 5.35 |
|  | **median** | 5.00 | 1.00 | 2.00 | 7.00 | 2.00 | 3.00 |
|  | **min** | 0 | 0 | 0 | 0 | 0 | 0 |
|  | **max** | 23 | 8 | 19 | 61 | 18 | 51 |
|  |  | **caregiver report** | | | | | |
|  | **mean** | 3.39 | 1.29 | 1.08 | 4.96 | 1.68 | 2.20 |
|  | **std** | 2.70 | 1.41 | 1.65 | 5.50 | 2.41 | 3.86 |
|  | **median** | 3 | 1 | 0 | 3 | 1 | 0 |
|  | **min** | 0 | 0 | 0 | 0 | 0 | 0 |
|  | **max** | 18 | 13 | 15 | 48 | 20 | 45 |
|  |  | **youth report** | | | | | |
| **4-year follow-up** |  | **number of total events** | **impact of total events** | **number of positively appraised events** | **impact of positively appraised events** | **number of negatively appraised events** | **impact of negatively appraised events** |
|  | **mean** | 5.29 | 1.47 | 2.42 | 8.54 | 2.62 | 4.62 |
|  | **std** | 3.60 | 1.39 | 2.31 | 7.20 | 3.11 | 5.10 |
|  | **median** | 5 | 1 | 2 | 7 | 2 | 3 |
|  | **min** | 0 | 0 | 0 | 0 | 0 | 0 |
|  | **max** | 24 | 10 | 17 | 50 | 20 | 40 |
|  |  | **caregiver report** | | | | | |
|  | **mean** | 3.72 | 1.38 | 1.19 | 5.56 | 1.89 | 2.43 |
|  | **std** | 2.98 | 1.57 | 1.74 | 5.97 | 2.74 | 3.96 |
|  | **median** | 3 | 1 | 1 | 4 | 1 | 1 |
|  | **min** | 0 | 0 | 0 | 0 | 0 | 0 |
|  | **max** | 22 | 11 | 17 | 48 | 23 | 42 |
|  |  | **youth report** | | | | | |
| **6-year follow-up** |  | **number of total events** | **impact of total events** | **number of positively appraised events** | **impact of positively appraised events** | **number of negatively appraised events** | **impact of negatively appraised events** |
|  | **mean** | 7.05 | 1.92 | 3.27 | 11.27 | 3.48 | 6.11 |
|  | **std** | 4.29 | 1.59 | 2.91 | 8.67 | 3.54 | 6.40 |
|  | **median** | 6 | 2 | 3 | 10 | 3 | 4 |
|  | **min** | 0 | 0 | 0 | 0 | 0 | 0 |
|  | **max** | 27 | 9 | 21 | 64 | 23 | 56 |
|  |  | **caregiver report** | | | | | |
|  | **mean** | 4.41 | 1.52 | 1.46 | 6.51 | 2.09 | 2.94 |
|  | **std** | 3.50 | 1.72 | 2.03 | 7.03 | 2.99 | 4.69 |
|  | **median** | 4 | 1 | 1 | 4 | 1 | 1 |
|  | **min** | 0 | 0 | 0 | 0 | 0 | 0 |
|  | **max** | 23 | 15 | 18 | 57 | 24 | 52 |

**Table S3.** T-tests contrasting number and impact of positively and negatively appraised events for the 2-year follow-up, 4-year follow-up, and 6-year follow-up timepoint for the youth report and caregiver report.

| **2-year follow-up** |  |  | **number of negatively vs. positively appraised events** | **impact of negatively vs. positively appraised events** |
| --- | --- | --- | --- | --- |
|  | **youth report** | **whole group** | t = 26.37, p < 0.001 | t = 32.14, p < 0.001 |
|  |  | **females** | t = 16.71, p < 0.001 | t = 22.00, p < 0.001 |
|  |  | **males** | t = 20.49, p < 0.001 | t = 23.43, p < 0.001 |
|  | **caregiver report** | **whole group** | t = -7.93, p < 0.001 | t = 9.08, p < 0.001 |
|  |  | **females** | t = -5.43, p < 0.001 | t = 6.23, p < 0.001 |
|  |  | **males** | t = -5.78, p < 0.001 | t = 6.60, p < 0.001 |
| **4-year follow-up** | **youth report** | **whole group** | t = 25.00, p < 0.001 | t = 23.69, p < 0.001 |
|  |  | **females** | t = 19.35, p < 0.001 | t = 19.06, p < 0.001 |
|  |  | **males** | t = 16.09, p < 0.001 | t = 14.51, p < 0.001 |
|  | **caregiver report** | **whole group** | t = -5.53, p < 0.001 | t = 7.89, p < 0.001 |
|  |  | **females** | t = -3.32, p = 0.0009 | t = 5.89, p < 0.001 |
|  |  | **males** | t = -4.48, p < 0.001 | t = 5.26, p < 0.001 |
| **6-year follow-up** | **youth report** | **whole group** | t = 23.07, p < 0.001 | t = 20.42, p < 0.001 |
|  |  | **females** | t =18.78, p < 0.001 | t = 18.25, p < 0.001 |
|  |  | **males** | t = 13.91, p < 0.001 | t = 10.42, p < 0.001 |
|  | **caregiver report** | **whole group** | t = -1.45, p = 0.1459 | t = 8.71, p < 0.001 |
|  |  | **females** | t = 0.22, p = 0.8225 | t = 6.84, p < 0.001 |
|  |  | **males** | t = -2.28, p = 0.0224 | t = 5.46, p < 0.001 |

**Table S4. Correlations depicting the overlap between youth and caregiver report**

|  | **Number of total events** | **impact of total events** | **Number of positively appraised events** | **Impact of positively appraised events** | **Number of negatively appraised events** | **Impact of negatively appraised events** |
| --- | --- | --- | --- | --- | --- | --- |
| **2-year follow-up** | r = 0.3175, p < 0.0001 | r = 0.3388, p < 0.0001 | r = 0.2369, p < 0.0001 | r = 0.2471, p < 0.0001 | r = 0.3007, p < 0.0001 | r = 0.3205, p < 0.0001 |
| **4-year follow-up** | r = 0.3147, p < 0.0001 | r = 0.3491, p < 0.0001 | r = 0.2483, p < 0.0001 | r = 0.2763, p < 0.0001 | r = 0.2588, p < 0.0001 | r = 0.3108, p < 0.0001 |
| **6-year follow-up** | r = 0.3604, p < 0.0001 | r = 0.3727, p < 0.0001 | r = 0.2707, p < 0.0001 | r = 0.2765, p < 0.0001 | r = 0.3093, p < 0.0001 | r = 0.3352, p < 0.0001 |

**
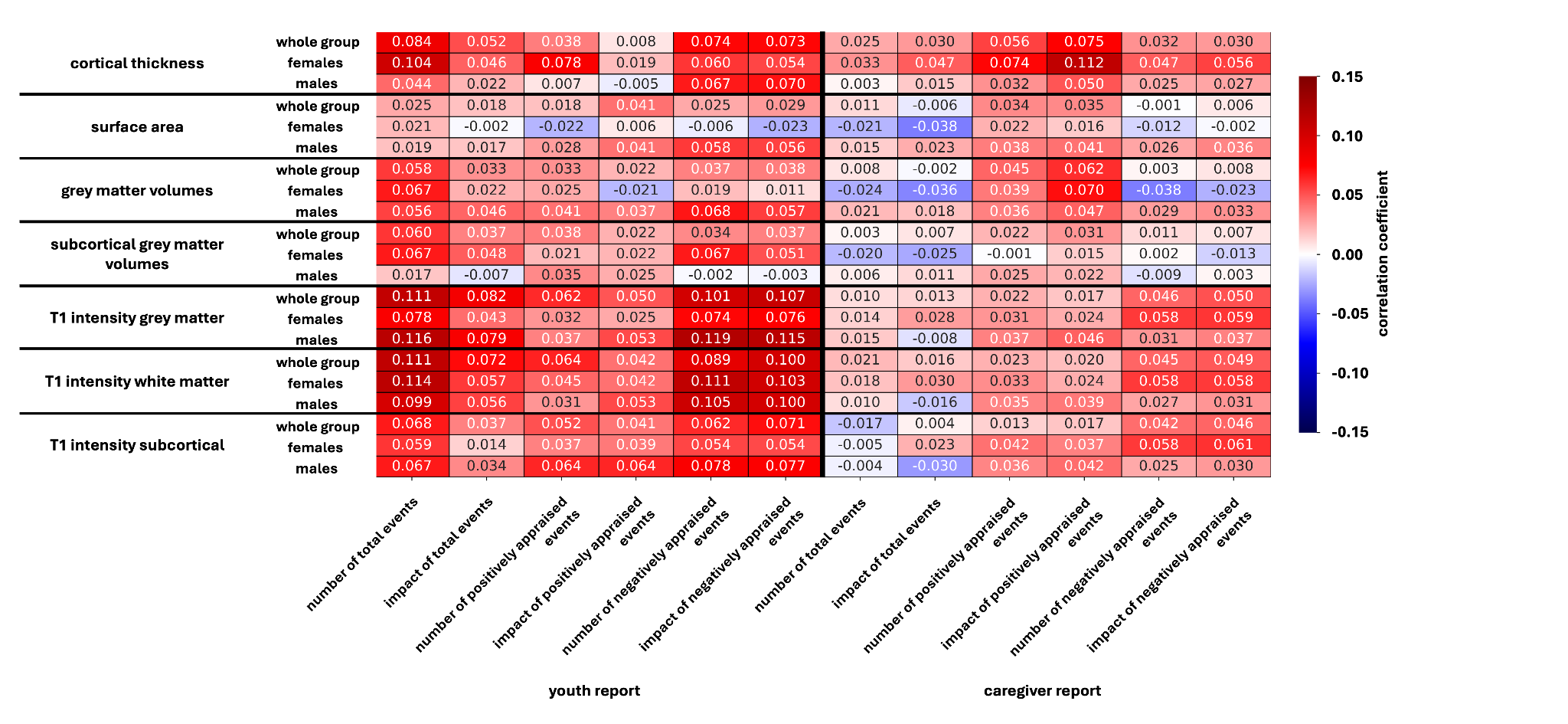
**

**Figure S4. Predictive Performance of neuroanatomical measures for stressful life event outcomes at 2-year follow-up.** This heatmap illustrates the mean Pearson correlation coefficients capturing the association between predicted and actual data for models trained to predict the number, appraisal, or impact of stressful life events based on different neuroanatomical measurements (Cortical thickness, surface area, cortical and subcortical grey matter volumes and T1-intensity measures). The colors of the heatmap indicate the strength of the association depicted by the mean correlation coefficient. Negative associations are depicted in blue, positive associations are depicted in red. Cortical measurements of cortical thickness, surface area, grey matter volumes and T1-intensity are based on the Destrieux parcellation.

**
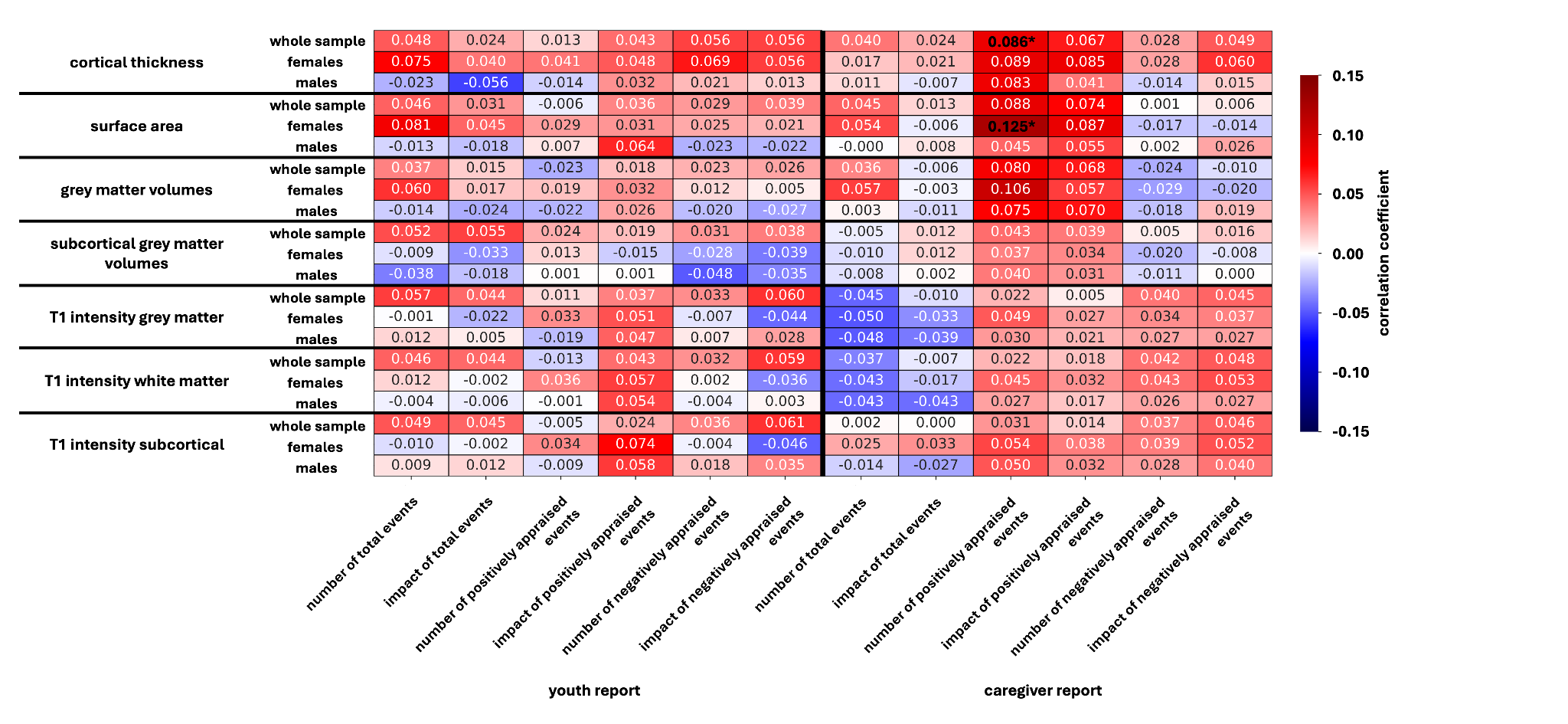
Figure S5. Predictive Performance of neuroanatomical measures for stressful life event outcomes at 4-year follow-up.** This heatmap illustrates the mean Pearson correlation coefficients capturing the association between predicted and actual data for models trained to predict the number, appraisal, or impact of stressful life events based on different neuroanatomical measurements (Cortical thickness, surface area, cortical and subcortical grey matter volumes and T1-intensity measures). The colors of the heatmap indicate the strength of the association depicted by the mean correlation coefficient. Negative associations are depicted in blue, positive associations are depicted in red. Cortical measurements of cortical thickness, surface area, grey matter volumes and T1-intensity are based on the Destrieux parcellation.

**
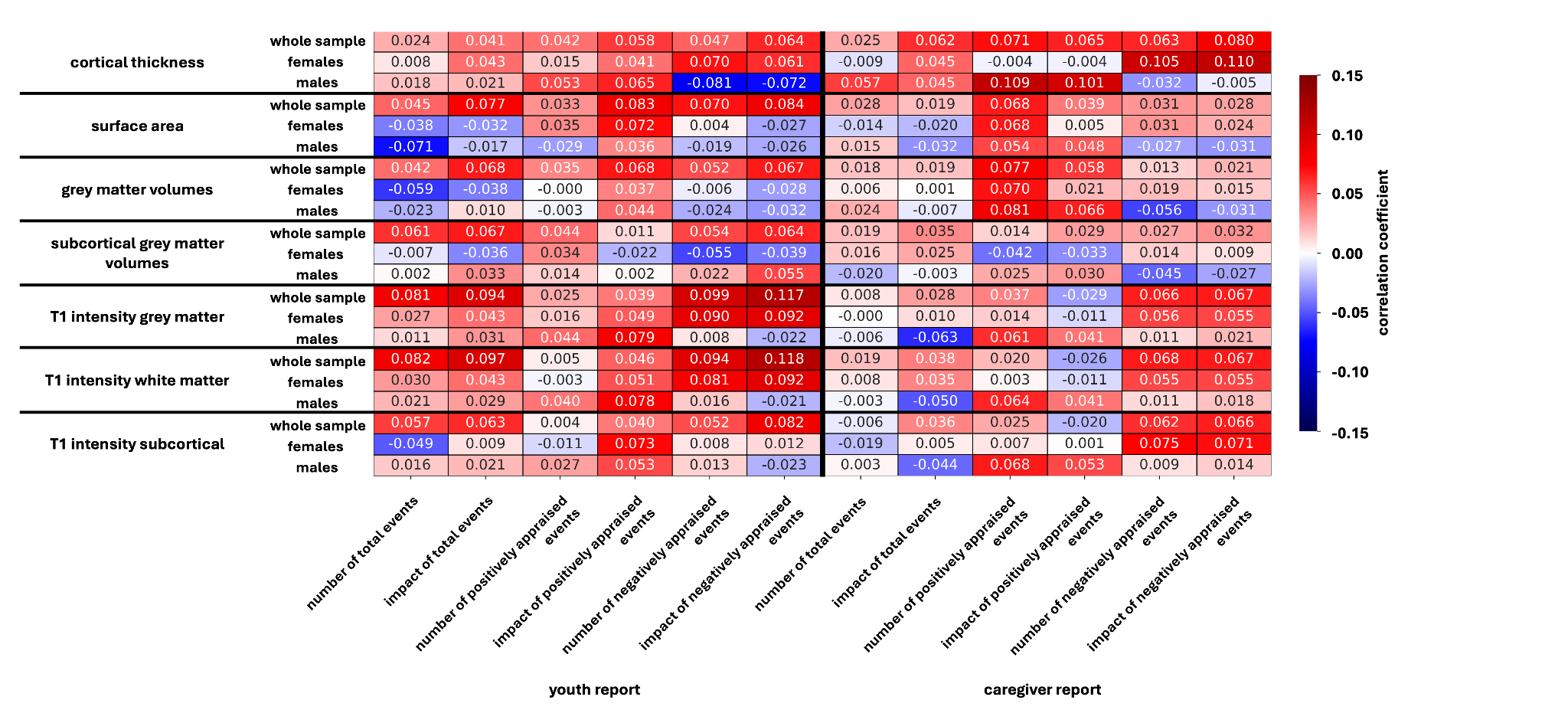
Figure S6. Predictive Performance of neuroanatomical measures for stressful life event outcomes at 6-year follow-up.** This heatmap illustrates the mean Pearson correlation coefficients capturing the association between predicted and actual data for models trained to predict the number, appraisal, or impact of stressful life events based on different neuroanatomical measurements (Cortical thickness, surface area, cortical and subcortical grey matter volumes and T1-intensity measures). The colors of the heatmap indicate the strength of the association depicted by the mean correlation coefficient. Negative associations are depicted in blue, positive associations are depicted in red. Cortical measurements of cortical thickness, surface area, grey matter volumes and T1-intensity are based on the Destrieux parcellation.


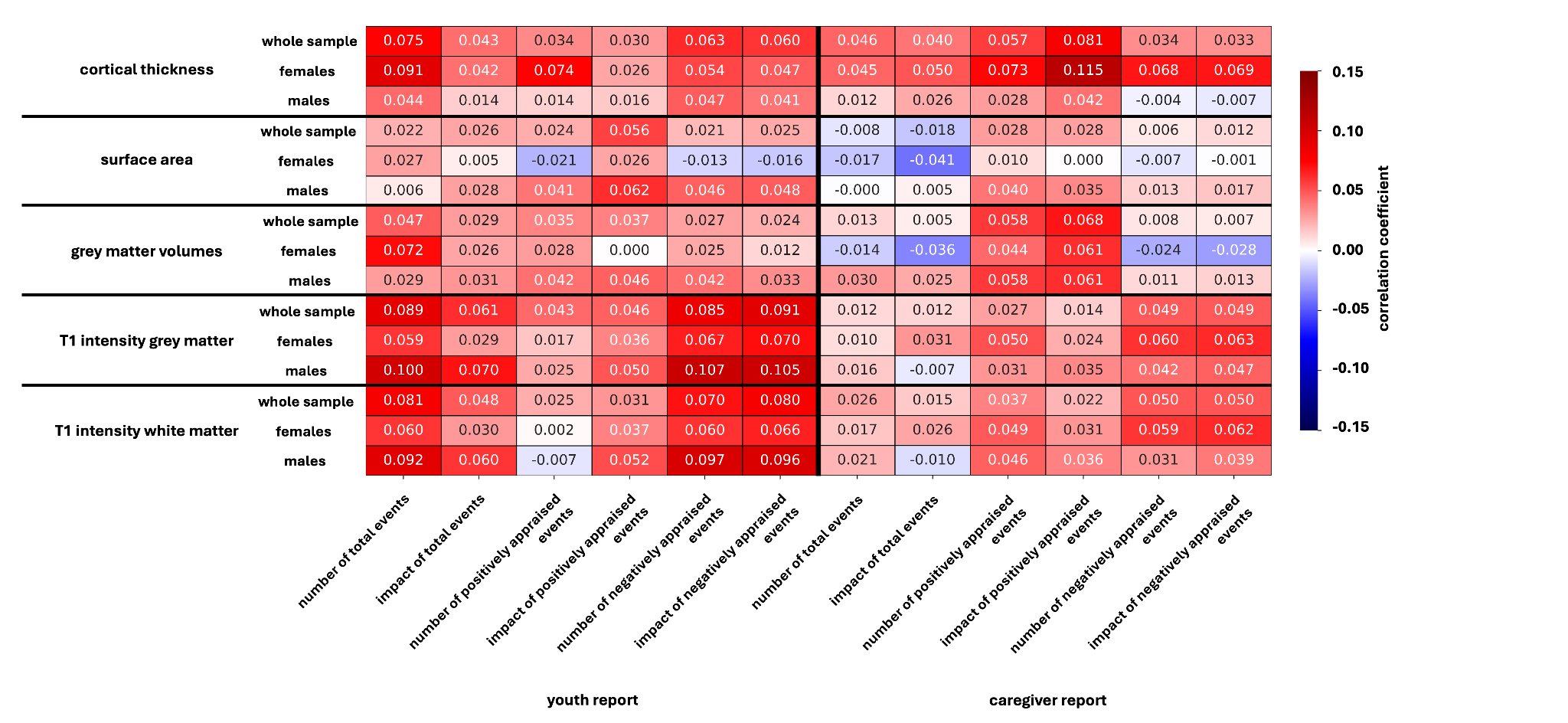
**Figure S7. Predictive Performance of neuroanatomical measures for stressful life event outcomes at 2-year follow-up.** This heatmap illustrates the mean Pearson correlation coefficients capturing the association between predicted and actual data for models trained to predict the number, appraisal, or impact of stressful life events based on different neuroanatomical measurements (Cortical thickness, surface area, cortical grey matter volumes and T1-intensity measures). The colors of the heatmap indicate the strength of the association depicted by the mean correlation coefficient. Negative associations are depicted in blue, positive associations are depicted in red. Cortical measurements of cortical thickness, surface area, grey matter volumes and T1-intensity are based on the Desikan parcellation.

**
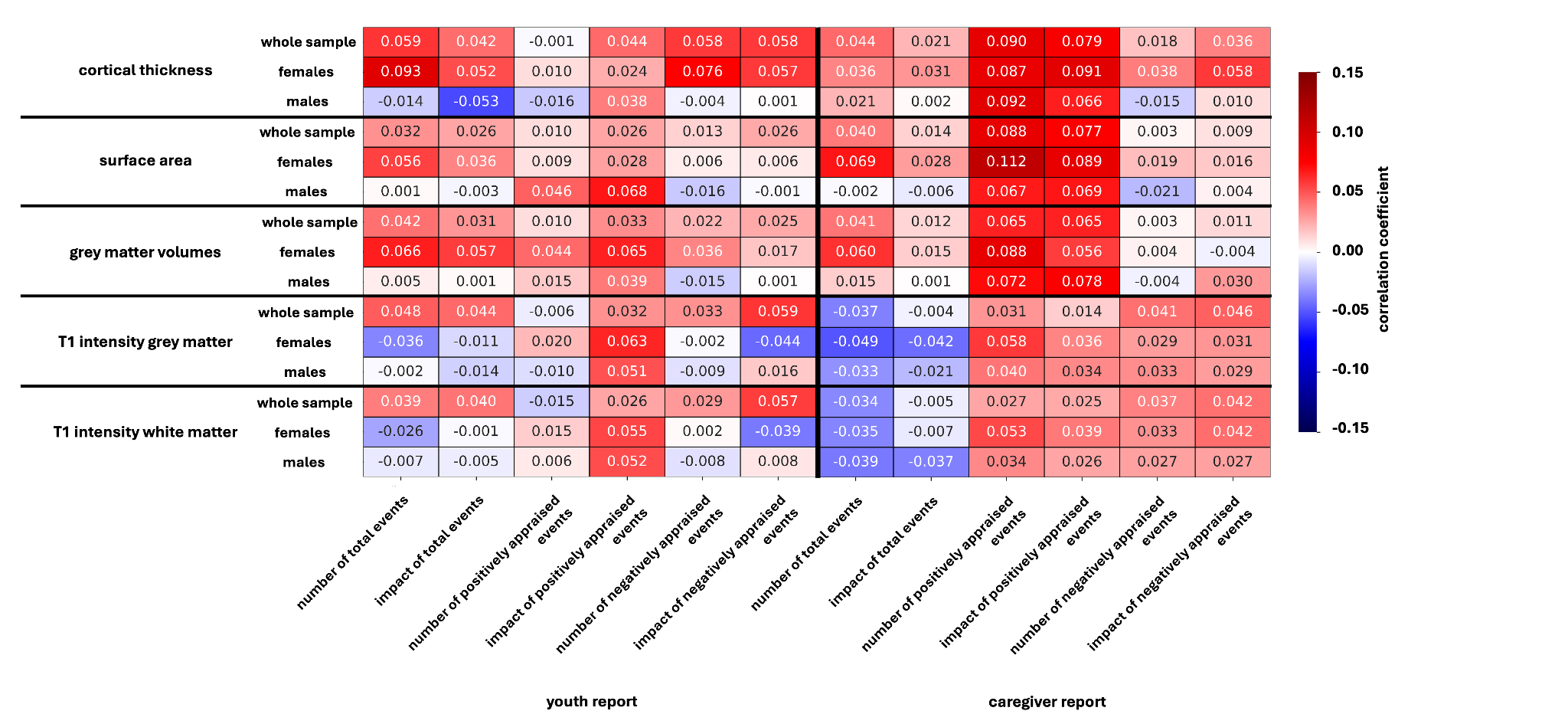
Figure S8. Predictive Performance of neuroanatomical measures for stressful life event outcomes at 4-year follow-up.** This heatmap illustrates the mean Pearson correlation coefficients capturing the association between predicted and actual data for models trained to predict the number, appraisal, or impact of stressful life events based on different neuroanatomical measurements (Cortical thickness, surface area, cortical grey matter volumes and T1-intensity measures). The colors of the heatmap indicate the strength of the association depicted by the mean correlation coefficient. Negative associations are depicted in blue, positive associations are depicted in red. Cortical measurements of cortical thickness, surface area, grey matter volumes and T1-intensity are based on the Desikan parcellation.

**
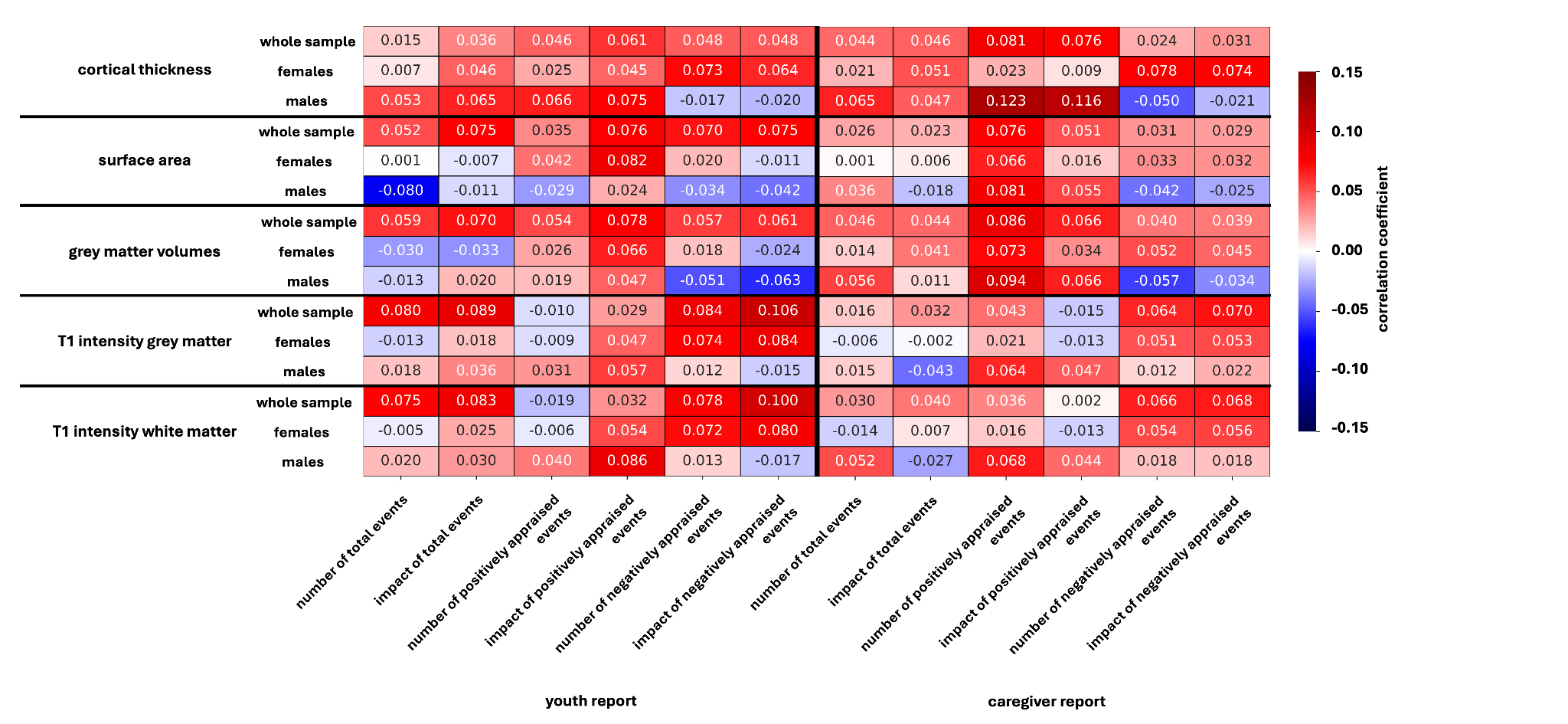
Figure S9. Predictive Performance of neuroanatomical measures for stressful life event outcomes at 6-year follow-up.** This heatmap illustrates the mean Pearson correlation coefficients capturing the association between predicted and actual data for models trained to predict the number, appraisal, or impact of stressful life events based on different neuroanatomical measurements (Cortical thickness, surface area, cortical grey matter volumes and T1-intensity measures). The colors of the heatmap indicate the strength of the association depicted by the mean correlation coefficient. Negative associations are depicted in blue, positive associations are depicted in red. Cortical measurements of cortical thickness, surface area, grey matter volumes and T1-intensity are based on the Desikan parcellation.
